# A Method of Preparing and Filling a Mesio-occlusal Cavity in an Upper Second Bicuspid

**Published:** 1902-03-15

**Authors:** E. K. Wedelstaedt

**Affiliations:** St. Paul, Minn.


### A METHOD OF PREPARING AND FILLING A MESIO-OCCLUSAL CAVITY IN AN UPPER SECOND BICUSPID.

BY DR. E. K. WEDELSTAEDT, ST. PAUL, MINN.

*Preparation*.—It is supposed that there is an adjoining tooth in position and that space has been obtained. That the teeth are normally situated and that the patient is eighteen or more years of age and in good health. It is also supposed that all burs, chisels and excavators are as sharp as it is possible to obtain or to make them.

Rapid work can be done only where there are sharp tools to be used and I believe in rapid work.

Study the occlusion and gain some adequate idea of the amount of stress the filling will be compelled to sustain. Unless this knowledge is obtained do not go on with the work. If the rubber dam has been adjusted prior to examining the occlusion, remove it and obtain the information that it is necessary to have, so as to deal with the case in an intelligent manner. Remember that the resistance and retentive forms of the cavity depend wholly upon the amount of stress the filling will be called upon to carry. Examine the occlusal surfaces of the other teeth and observe the condition of the cusps and note what use is made of the teeth.

Remove the temporary filling or separating material which has held the bicuspids apart. Wash the teeth and gums with warm water in which some oil of cassia has been placed (8 drops to the pint). Rinse the mouth with same kind of water throwing it in and around the teeth with some force—use a syringe for this purpose. Wash teeth and gums with disinfectant. especial care should be exercised to have the teeth and gums clean where dam will be placed and where it rests.

Adjust rubber dam. Personally, I prefer to expose the crowns of all the teeth from and including the first molars on the one end to and including the central incisor on the other end. Place ligatures around two bicuspids only. Secure dam and place small napkins under the holders to protect the face. Wash exposed crowns of teeth with alcohol, dry with air.

Outline cavity. Decide about where the cavity margins should be when cavity is finally prepared and ready for the gold. With a sharp chisel cut down lingual and buccal enamel margins, until they pass all contact with the adjoining tooth and until good sound tooth structure is encountered. Shape occlusal surface using the same chisel. If any decay remains in cavity remove it using a large spoon excavator. With an obtuse hoe excavator make a flat seat at the gingiva so that any filling will sit comfortably and not slide. With a fissure bur cut step in occlusal surface, just through the enamel. Increase the size of the step by using a sharp chisel. With a sharp chisel prepare cavity walls. With an inverted cone bur square up gingival seat and the occlusal step. With a good round-pointed obtuse hoe excavator cut two small pits in the gingivo-axial angles, not more than half a millimeter deep. With another sharp chisel prepare marginal form, plane it nicely. Inspect every part of the cavity, if it is satisfactory, note if a blast of cold air is disagreeable. If so, place a coat or two of varnish on and around the surface nearest the pulp (pulpal wall). Try air again.

What we should have from such procedures. A cavity whose lingual and buccal margins are perfectly straight from the occlusal surface to the gingival margin. The lingual and buccal margins would pass all con act with the adjoining tooth. When one is standing in front of the patient, the buccal margin should be perfectly visible and in looking into the mouth from the median line, the entire length of the lingual margin is seen, The cavity is large enough gingivo-occlusally so that any fil ing made in it will have the gingival portion of the filling covered with the gum septum. The gingival seat and occlusal step do not slope in any direction. They are perfectly flat. All the work up to this time should not have taken more than twenty minutes.

*Remarks.* The upper second bicuspids are about nine millimeters lingo-buccally, in thickness. The cavities which are made in their proximal surfaces, should, therefore, be in the neighborhood of six millimeters, lingo-buccally, provided permanent work is to be made. The reasons for this have so often been emphasized that it seems out of place to do more than to call attention to this fact.

*Filling.* It is optional with the operator where he begins his filling. He is at liberty to commence with annealed gold in the anchorage on the occlusal surface or he can begin placing his unannealed foil in the gingival third of the cavity on the proximal surface. *Under no circumstances is a matrix ever to be used where gold fillings are made.*

Let us begin by placing our gold in the gingival third of the cavity. An unannealed cylinder, containing one quarter of a sheet of No. 4 gold foil, is first placed in one of the shallow pits made in the gingivo-axial angle. This is partly condensed. Another unannealed cylinder of the same size is next placed in the opposite gingivo angle. This is also partly condensed. Another unannealed cylinder containing a half a sheet of gold is now placed between the two previously inserted, and very often still another half sheet is used. The last piece or pieces used act as a wedge and thus force the first two pieces into their positions and hold them there. That portion of the gold lying against the pulpal wall is now condensed.

The gold is now annealed and placed into the anchorage made on the occlusal surface. Sufficient gold is built into the anchorage and across the step to have a good strong mass of material. This mass of material is extended constantly until the annealed gold is packed over that portion of the unannealed gold previously placed into position in the gingival third of the cavity.

As soon as the annealed gold has been packed over the condensed unannealed gold, the condensing of the remainder of the unannealed gold, is, step by step, proceeded with until it is fully condensed. The gold is laminated backwards and forwards, from the anchorage in the occlusal step to and over the first three or four pieces placed in the gingival third of the cavity until the cavity is overfull. The gold for the contact point receives more condensation than other parts of the filling. As soon as the gold has all been placed, strong marginal condensation follows.

*Finishing Filling.* At the completion of the marginal condensation, the surplus gold around the gingival margin is removed. A very thin narrow saw in a Black saw frame is used for this purpose. The Black files and trimmers are now used to trim and contour the proximal portion of the filling. The occlusal portion can be trimmed with the finishing burs or stones. The polishing of the filling is accomplished with strips of No. oo Emery cloth, pumice stone, chalk, rubber points, brushes, etc.

Under no circumstances are rotary discs to be passed between the proximal portion of the filling and the adjoining tooth. They can, however, be used to finish that portion of the filling to the buccal or lingual of the contact point or on the occlusal surface, but they should not be used otherwise. If they are passed between the teeth, the contact point is destroyed and a proper contour of the interproximal space is not obtained. I am aware that a number of dentists are constantly asserting that they do all their finishing with rotary discs and strips. I should judge that such was the case from the appearance of their finished operations. Such operators do not know what constitutes a proper interproximal space, or if they do know, prefer to ignore it.

*Coutour of the interproximal space.* We may know when the interproximal space is contoured properly—reference is now made to the operation described in this essay—by drawing the cheek to one side and then asking the patient to close the mouth and then raise the tongue against the bicuspids. If the saliva readily runs through the gingival space, the work has been properly made. If the saliva does not run through the space, when the tongue is raised, the work has not been properly made. I am constantly seeing many beautiful fillings which are unusually made but they are not contoured properly. Such operations are a menace to health and to the integrity and stability of the rest of the teeth on account of their ability to invite disease. It is our duty to correct these evils and men of knowledge, who have the welfare of their patients at heart, correct them. Contouring properly the interproximal space is not a theory as so many seem to think, but it is a condition which must receive much consideration from all who desire to make the highest grade of dental work. Gold and porcelain crowns are included in this category. It is as necessary to give them proper contour as it is to contour fillings properly.

It should be our aim and we should constantly endeavor to so finish all fillings made in the proximal surfaces of teeth, that they will be free from pits, depressions, etc. For many reasons fillings should be given a "looking glass" finish.—*Dental World.*

